# The Impact of Postures and Moving Directions in Fire Evacuation in a Low-Visibility Environment

**DOI:** 10.3390/s24051378

**Published:** 2024-02-21

**Authors:** Jingjing Yan, Gengen He, Anahid Basiri, Craig Hancock, Siegfried K. Yeboah

**Affiliations:** 1 International Exchange College, Ningbo University of Technology, Ningbo 315211, China; 2 International Doctoral Innovation Centre, University of Nottingham, Ningbo 315100, China; 3 Department of Geographical Science, University of Nottingham, Ningbo 315100, China; gengenhe@gmail.com; 4 School of Geographical & Earth Sciences, University of Glasgow, Glasgow G12 8QQ, UK; ana.basiri@glasgow.ac.uk; 5 School of Architecture, Building and Civil Engineering, Loughborough University, Loughborough LE11 3TU, UK; c.m.hancock@lboro.ac.uk; 6 School of the Built Environment and Architecture, London South Bank University, London SE1 0AA, UK; yeboahs5@lsbu.ac.uk

**Keywords:** building fire safety, building fire evacuation, evacuation time, evacuation speed, stoop walking

## Abstract

Walking speed is a significant aspect of evacuation efficiency, and this speed varies during fire emergencies due to individual physical abilities. However, in evacuations, it is not always possible to keep an upright posture, hence atypical postures, such as stoop walking or crawling, may be required for survival. In this study, a novel 3D passive vision-aided inertial system (3D PVINS) for indoor positioning was used to track the movement of 20 volunteers during an evacuation in a low visibility environment. Participants’ walking speeds using trunk flexion, trunk–knee flexion, and upright postures were measured. The investigations were carried out under emergency and non-emergency scenarios in vertical and horizontal directions, respectively. Results show that different moving directions led to a roughly 43.90% speed reduction, while posture accounted for over 17%. Gender, one of the key categories in evacuation models, accounted for less than 10% of the differences in speed. The speeds of participants under emergency scenarios when compared to non-emergency scenarios was also found to increase by 53.92–60% when moving in the horizontal direction, and by about 48.28–50% when moving in the vertical direction and descending downstairs. Our results also support the social force theory of the warming-up period, as well as the effect of panic on the facilitating occupants’ moving speed.

## 1. Introduction

Buildings occasionally face hazards such as fire events which can threaten life, building structure, property, and environment [1]. According to Kobes et al. [2], in a fire event involving a building, the most crucial aspect is the possibility of a safe escape for the occupants. More importantly, in a building, the fire safety facilities should enable independent and adequate fire response performances by the evacuees [2]. For instance, Arewa et al. [3] argues that evacuation strategies such as the stay-put tactic could be beneficial to protect, control, and facilitate the smooth evacuation of occupants during fire incidents, or could be a misjudgement and a futile strategy with potential fatalities, such as in the 2017 Grenfell Tower fire event in London, England.

Evacuee behaviour resulting from building evacuation strategies plays an important role in a performance-based design for fire safety, hence, its understanding and prediction can help improve the safety guidance for buildings [4,5]. With the development of evacuation software, more egress models with powerful capabilities are available. The accuracy of simulations using such software becomes even more crucial as the data on the characteristics of occupants, their actions during evacuation, delays that may occur, and travel speeds for different types of occupants depicting evacuee behaviours become a critical requirement [6]. These data are relatively deficient with a limited amount of fire events and experiments [4,7,8,9]. According to Fahy and Proulx [6] and Ronchi [10], data collection for evacuation models can generally be divided into six categories, as follows:

- Pre-movement time, i.e., the time period between alarm triggering and the beginning of occupant movement.
- Movement speeds in horizontal (moving in corridors) and vertical directions (moving in staircases) [11,12].
- Occupant characteristics, including all factors affecting the actions and responses of different types of occupants, e.g., age, gender, training degree, etc. [13,14].
- Occupant decisions on actions during the evacuation process [15].
- Delay or block effects occurring due to route availability and flow constraints, such as obstructions [11,16,17].
- Exit choices [15].

According to the Occupational Safety and Health Administration (OSHA), occupants should evacuate buildings as fast and safely as possible in an emergency like a fire event [18]. It is important to point out that the strong demand to escape through the corridors and exit gates during a fire event in a building have been found to result in congested flow, often leading to casualties and a drop in evacuation efficiency; hence, optimum schedules for staged-evacuation processes have been advocated [19]. Hosseini et al. [19], for instance, outlined heterogeneous objectives that take into account the total evacuation time (TET), the threat of fire exposure, and the congestion severity. The TET is usually divided into two parts: the pre-movement time and the travel time [20]. Pauls [21] argued that predicting minimum evacuation times, even realistic minima, should be performed carefully regarding the input assumptions (on flow, speed, etc.), due to the complications of human behaviour in an emergency such as a fire.

In determining the total evacuation time, pedestrian speed, regarded as a key element, represents people’s physical abilities [14,22,23]. It plays an important role in the calculation of the travel time via the quantifying of occupant movements [24]. The walking speed and travel time of occupants have been investigated for more than five decades, including some major research on movement across the evacuation path from Fruin, Predtetschenski, and Milinski [2,6,22,25], Habicht and Braaksma [25], and Yamada and Akizuki [26]. Many of the previous studies focused on the relationship between movement speed and occupant characteristics, such as gender [23,27,28], body mass index (BMI) [23,24], and the psychological state [29,30,31]. These studies collected data under non-emergency and emergency conditions [32], which included movements in horizontal directions. They assumed an erect walking posture, and a walking velocity of 1.34 m/s under non-emergency conditions [27,33,34]. During emergencies, the walking speed increased beyond the speed under non-emergency situations. For example, Muhdi et al. [22] measured the average walking speed, reporting an average of 1.32 m/s under non-emergency conditions, but found it could increase to a maximum of 2.16 m/s during emergencies. Zhao et al. [30] also recorded average walking speeds of 1.32 m/s under normal conditions, as well as a maximum of 2.91 m/s during emergencies. 

However, it is not always possible for people to keep an upright walking posture during evacuation. Harmful by-products of the combustion process of a fire event, such as heat, smoke, and burning gases, may require an atypical posture (such as stoop walking or crawling) for survival, rather than an upright pose [22,23,27,33]. Compared to the number of studies on up-right walking during a fire evacuation, limited studies have investigated the moving speed of evacuees using atypical postures [32]. Most of the studies found in the literature concern crawling postures [22,23,24,28,33,34,35], while there are even fewer studies on evacuating with a stoop posture [28,34,35]. 

From the literature, crawling was found to impede the speed of evacuation, as it significantly reduces the speed of movement. Several studies reported a reduction in crawling speed by about 36.8–66.7% [22,23,27,28,33,35], and the speed of crawling to be in a range between 0.5 m/s and 0.87 m/s [22,23,24,27,28,33,34,35,36]. With the average walking speed during evacuation being reported as 1.32 m/s, the maximum speed of 0.87 m/s when crawling represents around 65% of the speed as when evacuees are walking to safety. On the other hand, the speed reduction of stoop-walking compared to upright walking is much less than that of crawling. Here, a maximum reduction of 24% is reported when moving under conditions of low heights, typically <1.2 m [35], and bending more than 70% of the upright posture [36]. For situations without severe bending, the speed reduction was found to be in a range between 4.66% and 11% [27,28]. As crawling requires more metabolic energy consumption (73~375%) than upright walking [27,28,34,36,37], occupants will likely suffer from fatigue when crawling for longer distances, leading to relatively low survival rates (4.17~16.67%) [28]. These facts make crawling not an ideal option for evacuation [22,34]. 

In addition, the stoop posture is more likely to happen when moving in a low-visibility environment as it can help identify frontal situations [38]. Many previous studies have also investigated walking speeds under low-visibility conditions, indicating visibility as an important affecting factor, especially under extreme conditions of less than 3 m of visibility [38,39,40,41,42,43,44,45]. According to the previous data, the speed can reduce from 2.03 m/s to 1.74 m/s, or even as low as 1.28 m/s with less than 1 m of visibility [41,45]. Under some extreme conditions, the speed could be even slower than 0.62 m/s when the visibility is near-zero [42,43]. However, these studies did not shed light on the postures applied during the experiments or the degree to which participants lowered their heights. Nonetheless, the experimental details appear to show that upright postures were used. In this present study, we introduce two different types of stoop postures for evacuation, i.e., trunk flexion only and trunk–knee flexion, both with height reductions of 30% compared to the reference upright posture. This is because the degree of bending over this threshold potentially increases the risk of falling and thus impedes movement [46,47]. 

In previous studies, the walking velocities using different postures were usually measured with a professional set of cameras and/or electromyography (EMG) equipment [27,28,34,35,48]. These methods either require more than ten fixed cameras at the test site [9,20,34,35,38,40,48,49,50,51,52], or a self-developed equipment set with attached cameras [27,28,32,33,53]. However, this kind of method may not be functional when the cameras are covered by the smoke of a fire event, and thus may only be used under experimental conditions without vision impediment. Also, large storage requirements for the video data may be a limitation. For this reason, in this study, a self-designed indoor tracking system, i.e., a 3D passive vision-aided inertial system (PVINS) [54], capable of working in good or poor visibility environments, as it utilises vision-calibrated smartphone-embedded inertial sensors, was used. The PVINS does not rely on stored video data for its analysis as it acquires inertial data, which requires less storage and is more suitable for real-time monitoring. This is regarded as one of the major novelties of this study as it provides a supplementary method for visual tracking in invisible areas. 

Additionally, previous studies reviewed involved mainly only using a single posture for each evacuation test. However, in a real fire event, multiple postures may be used along the evacuation path under changing environmental conditions. To mimic conditions as realistically as possible, our investigation focused on different postures during evacuation under emergency and non-emergency scenarios. The novelty in our study is the use of mixed postures during the evacuation process, used realise the potential differences in speed.

The literature also shows limited investigations in studies focused on evacuation movements in a vertical direction. Previous studies reviewed tested vertical moving speeds on staircases with different thread and riser ratios, and found typical vertical movement speeds in the range of 0.62–1.25 m/s, depending on evacuee characteristics [51,55,56,57]. Other researchers have investigated moving speeds under varying visibilities, and found the mean vertical velocity of evacuees to range between 0.41 m/s and 0.57 m/s; while under 100% illumination, speeds ranged between 0.85 m/s and 1.25 m/s [39,49,50,51,58,59]. Only a few previous studies [39,49] have involved two moving directions in one track; however, they focused on the impacts on the ascending speed, as they executed evacuation experiments in an underground site. In this study, we investigate how different postures, such as a stooped posture and an upright posture, affect walking speed when evacuees are escaping in the vertical (descending) and horizontal directions, respectively. Here, the third contribution is that we evaluate a mixture of evacuation design scenarios with different postures under emergency and non-emergency conditions in a low-visibility environment.

In this paper, we firstly introduce a novel method which covers the invisible areas of the tracking cameras with compensable inertial data, rather than the vision-only method used in previous studies. Secondly, we use two specific stoop postures applied for fire evacuation, as opposed to the conventional upright, crawling, or unstructured and unspecified postures in other studies. Furthermore, we introduce a variety of different postures and combinations of postures during our experiment. Finally, we investigate evacuations using these postures under varied scenarios, utilising both horizontal and vertical moving directions under non-emergency and emergency situations. In our review of the literature, we found previous studies which mainly focused on only one kind of situation (emergency or non-emergency) and one moving direction. 

The rest of the paper is organised as follows: Section 2 focuses on materials and methods, discussing the experimental design and measurement methodology. Section 3 outlines the experimental results and the comparison with previous studies, while Section 4 provides the analyses of the potential effects of different factors on walking speeds, as well as a discussion of the limitations of the study. Finally, the conclusion, provided in Section 5 outlines the key findings and recommendations for future investigations. 

## 2. Materials and Methods

### 2.1. Volunteering Participants Characteristics

Twenty volunteering participants, comprising ten female and ten male undergraduate students between 21–23, consented to partake in the study after completing a faculty ethics panel-approved consent form. The participants had a body mass index (BMI) in the range of 19.9–24.2. The female participants had a height in the range of 160–165 cm and a weight in the range of 50–55 kg. The heights of the male participants ranged between 175–180 cm, and their weights ranged between 65–75 kg. The sample size was satisfactory, given that previous studies utilised a sample size of a minimum of nine participants [23,27,28,33,34].

### 2.2. Equipment

**Figure 1:**
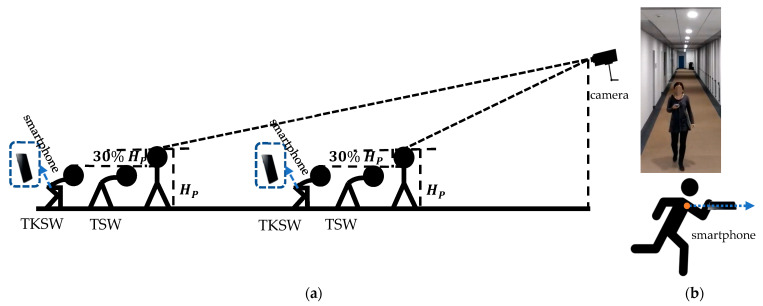
The experimental setup of cameras for upright walking, trunk flexion stoop walking (TSW), and trunk–knee flexion stoop walking (TKSW) ($H_{P}$ represents body height of the subject) (**a**), and the position of the smartphone held by the participants during movements in both the conceptual and site scenes (**b**).

A 3D PVINS, a vision-assisted system for indoor positioning, was used to measure the specific velocities of participants’ postures during the experiment [54]. The equipment was set up indoors for horizontal and vertical movement tracking, with an accelerometer sampling rate of 50 Hz, a frame detection rate of 17 Hz, and the pressure data of 1 Hz. The detailed setup was similar to that in Yan et al. [54] (See Figure 1), with surveillance cameras directly facing the corridors, and with smartphone-based inertial sensors held by the participants. 

The movements of the participants were tracked via embedded sensors in smartphones which were held in front of the participant’s chest, facing the walking direction. Simultaneously, participants were filmed by the surveillance camera system inside the building for the accelerometer calibration when moving horizontally. The vertical movements did not involve the cameras, using instead the vision-calibrated inertial sensor and smartphone-embedded barometer. Both the camera and barometer were self-calibrated before the experiments. The details of the sensors and their accuracies can be found in Table 1, and the methods of the sensor calibration can be found in [54].

### 2.3. Test Building Layout and Experimental Setups

**Figure 2:**
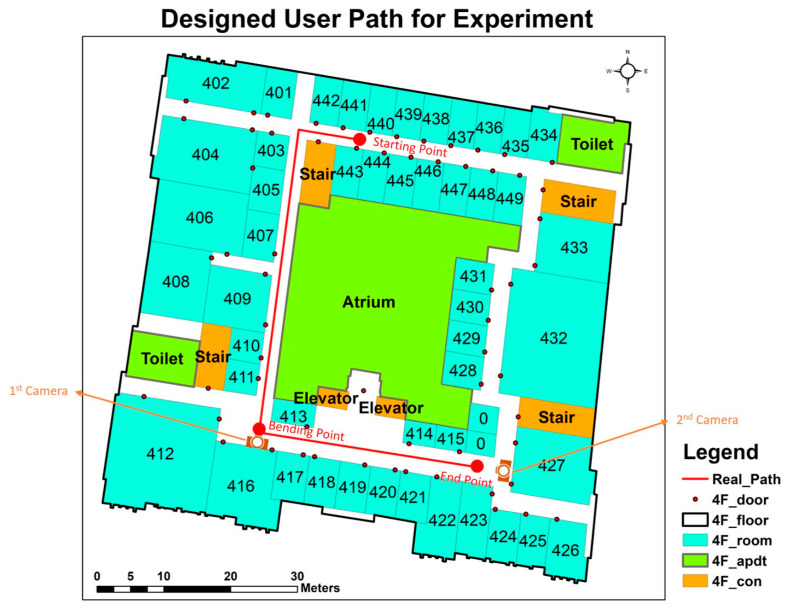
The route for the horizontal movements with the locations of the cameras (where ‘adpt’ represents rooms other than offices, and ‘con’ represents stairs and elevators).

**Figure 3:**
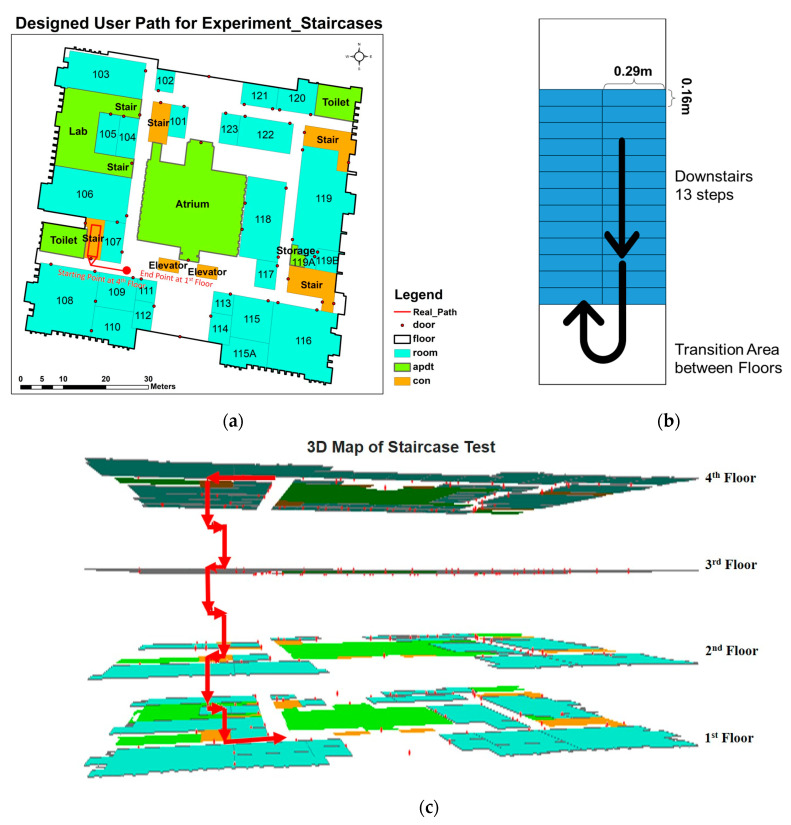
The test route for the vertical movements from the 4th floor to the 1st floor in 2D (**a**) (where ‘adpt’ represents rooms other than offices, and ‘con’ represents stairs and elevators), the schematic image of the stairs’ structure (**b**), and test route in 3D format (**c**).

The test building was a four-floor building at the University of Nottingham Ningbo, China, with a full surveillance system installed inside. The cameras were all about 3 m above the floor, positioned to capture the movements in the corridors. The entire track length for horizontal movements was about 92.75 m long (see Figure 2, Table 2), and the height for vertical movement was about 13.07 m high (see Figure 3, Table 2). Markers indicating the starting, bending, and end points were marked on the floor to guide participants. During the movement stage, the indoor environment was lit at an illuminance of 245 lux [50]. To simulate poor visibility, participants were made to wear glasses with a 10% visible light-transmission rate, similar to that used in Zeng et al. [51]. This was closer to the lights-off condition with reduced emergency lighting (≈74 lux), although in previous studies [50,51], 0% illumination refers to all lights off with only photoluminescent material (PLM). 

There were two scenarios applied in this study: non-emergency and emergency scenarios. These scenarios were distinguished based on the instructions given to the participants. Under non-emergency scenario, participants were instructed to walk normally, while under emergency scenarios, they were asked to walk as in an actual fire emergency as quickly as possible. For trials of either horizontal or vertical movements, participants were required to complete the non-emergency scenarios first, and then the emergency scenarios. The horizontal experiments were conducted first for vision-aided inertial sensor calibration. The calibrated inertial sensors were then used with barometers for vertical movements. The details of the experimental setups for the specific postures applied in the different scenarios can be found in Table 2.

**Table 2:** Experimental setup for movements.

Direction	Participants	Scenario	Posture	Experimental Space	Distance (m)	Illumination (%)
Horizontal	20 (10 M/10 F) age: 21–23 BMI: 19.9–24.2	Non-Emergency Scenario	Upright	Corridors of the 4th floor of building	92.75	10
		Emergency Scenario	Upright and Trunk Flexion		60.9 (upright), 31.85 (stoop)	
			Upright and Trunk Knee-Flexion			
Vertical		Non-Emergency Scenario	Upright	Corridors and Staircases from 4th floor to 1st floor in the building	13.07 (height), 76.45 (length in 2D)	
		Emergency Scenario	Upright			

### 2.4. Walking Postures and Acoustic Signals

When moving in horizontal directions, there were three postures applied: the upright posture, the trunk flexion stoop posture, and the trunk–knee flexion stoop posture. The stoop postures required a 30% reduction of body height $H_{P}$ (Figure 1). This was based on the previous findings of the maximum available height reduction for long-term stable bending postures for females [36]. 

During the experiment, each participant moved on hearing the instructor’s acoustic signal ‘Start!’. Participants kept an upright posture until the end point during the non-emergency scenario. During the emergency scenario, participants were instructed to bend at the bending mark in order to change from upright to the relevant stoop posture for the trial. The vertical movements did not involve bending postures, as the fall risk was estimated to be relatively high when moving downstairs in the stoop posture [46,47]. Some volunteering participants also objected due to concerns for their own safety. Therefore, the measurements of walking speeds both up and down the staircases only focused on the use of the upright posture.

The applied stoop posture during the emergency call was evenly chosen by the instructor during the experiment. For example, if one subject was instructed to move with the trunk flexion posture, the next subject was instructed to use the trunk–knee flexion posture. Volunteering participants could rest if they preferred before conducting another trial.

### 2.5. Measurement of the Horizontal and Vertical Velocity

#### 2.5.1. Measurement of Horizontal Velocity

Before the measurements were taken, the volunteering participants were trained to operate and familiarise themselves with the inertial data recording apps on the testing smartphones. The acquired accelerations of the detected steps were later utilised to calculate the velocity of each subject. The volunteering participants were trained to use the upright and stooped postures (Figure 1), and to react under different acoustic signals mentioned in Section 2.4. In this experiment, each participant was required to walk through a 92.75 m-long corridor, located on the fourth floor of the test building (Table 2). The starting and end points, as well as the bending points, were marked on the floor for each volunteering participant to step on when the instructor gave the relevant signal (Figure 2). The walking posture combinations investigated were upright and trunk-only flexion, and upright and trunk–knee flexion, respectively. 

The volunteering participants were required to wear glasses with approx. 10% light transmission in order to simulate low-visibility in a fire event (Section 2.3). During the measurements, the volunteering participants were asked to turn on their inertial data recording at the starting point. Simultaneously, the video recording commenced. As part of the preparations, participants waited for about 50 s while listening to the instructions from the recorder. On hearing the starting signal, each participant assumed an upright pose. Under the non-emergency scenario, the volunteering participants walked normally to the end point. Under the emergency scenario, each participant used an erect posture to move forward until the bending point, which was 60.9 m from the starting point. The bending acoustic signal was given when subjects stepped on the bending point. The rest of the evacuation simulation was then completed in a stoop posture in the form of either trunk flexion or trunk–knee flexion, based on the given instructions (Section 2.4). Each volunteering participant repeated the trials under non-emergency and emergency scenarios four times, respectively.

#### 2.5.2. Measurements of Vertical Velocity

The measurements of the vertical movements were conducted on the same day, following the measurements of horizontal movement velocities. Further training using the pressure data collection app, Barograph, was provided for each volunteering participant during their rest period after the horizontal measurement. During the vertical measurement, each volunteering participant was required to move downstairs from the fourth floor to the first floor (Figure 3), covering a track length of 76.45 m, with 13.07 m of height (Table 2). The number of stairs between each layer was 26, with 13 steps on one side, and the tread and rise of all stairsteps were 0.29 m and 0.16 m, respectively (Figure 3c). 

Each volunteering participant was required to turn on the inertial and barometer data recorder at a marked point on the 4th floor, and then waited for about 40–50 s before descent. Both the smartphone-embedded barometer and the inertial readings were recorded to calculate the velocity of the movements [54]. Like the horizontal measurements, the scenarios under the emergent and non-emergent conditions were distinguished through different instructions. On hearing the starting signal, each volunteering participant moved from the starting point and walked downstairs in an upright posture to the end point on the first floor (Figure 3). Under a non-emergency call, each volunteering participant moved normally, while under an emergency call, they moved with urgency as in a real fire event. The trials were repeated four times under non-emergency and emergency call scenarios, respectively. In between trials, the volunteering participants rested before the next measurement.

### 2.6. Data Logging and Processing

During the experiment, the testing smartphones were provided by the instructor. The smartphone-based inertial sensor data, i.e., the accelerations and angular velocities, were recorded using the data-recording app, MATLAB Mobile. The pressure data used for height change detection were simultaneously obtained with the inertial data using another smartphone app, Barograph. The camera data were recorded and collected anonymously for the horizontal movements, without storing any personal information, i.e., ‘participant 1, participant 2, …’, etc. The video data were deleted after extracting the participants’ positions. The processed video data, together with the smartphone-based data, were then stored on the storage disk and managed by the university without being shared. The recording time for the horizontal movements was about 150 s, and for the vertical movements, it was about 200 s, including the preparation period (for removing sensor noises and listening to instructions) and the moving period.

During the horizontal movements, each camera focused on monitoring one corridor and the collected data, processed using Faster R-CNN for participant detection. The captured frames that were too far or too close to the camera were removed in order to improve the detection accuracy. The calibrated inertial sensors were used to record participants’ step lengths. The instantaneous velocities $v_{h}\left(i\right)$ in the horizontal direction were then acquired by dividing each step length ${SL}_{i}$ by its time interval t(i)−t(i−1), as shown in Equation (1):

(1) $$v_{h}\left(i\right)=\frac{{SL}_{i}}{t(i)-t(i-1)}\left(i=1,2,\ldots ,n\right)$$

The details of processing all the above-mentioned data can be found in Yan et al. [54]. The vertical instantaneous velocity $v_{v}\left(i\right)$ on the staircases was then calculated by dividing the integrated step length ${SL}_{i}$ and stair riser by its time interval t(i)−t(i−1), as shown in Equation (2):

(2) $$v_{v}\left(i\right)=\frac{\sqrt{{{SL}_{i}}^{2}+{riser}^{2}}}{t(i)-t(i-1)}\left(i=1,2,\ldots ,n\right)$$

This only works on the staircases with changing heights. As the whole process of walking down from 4th floor to the 1st floor also involves some horizontally moving distances, these velocities were divided into two parts and calculated independently. This was completed by checking height differences from the recorded pressure data based on the method mentioned in [54]. Where there were significant height changes, the velocity was processed based on Equation (2), otherwise, it was based on Equation (1). After acquiring these instantaneous values, Equation (3) was used to automatically recognize any sudden changes of the average speed at different stages and corresponding directions.

(3) $$\left\{\begin{array}{c} \bar{v_{h}}=\frac{1}{n_{j}}\sum_{i=1}^{n_{j}}v_{h}\left(i\right)\\ \bar{v_{v}}=\frac{1}{n_{k}}\sum_{i=1}^{n_{k}}v_{v}\left(i\right) \end{array}\right.$$

where $n_{j}$ and $n_{k}$ represent the corresponding steps of the specific stages (Stage j or Stage k) in different walking directions, and $\bar{v_{h}}$ and $\bar{v_{v}}$ represent the average value in horizontal and vertical directions, respectively. 

The average speed $\bar{v}$ changes $P_{d,p,s,g}$ are calculated using Equation (4):

(4) $$P_{d,p,s,g}=\frac{{\bar{v}}_{d,p_{m},s_{,}g}-{\bar{v}}_{d^{\prime },\;\;p_{m}^{\prime },s^{\prime },g^{\prime }}}{{\bar{v}}_{d^{\prime },p_{m}^{\prime },s^{\prime },g^{\prime }}}$$

where d stands for two moving directions as horizontal and vertical directions, $p_{m},$ stands for different postures (m contains upright, trunk flexion, and trunk–knee flexion), s stands for two different scenarios (non-emergency and emergency), and g stands for gender. 

## 3. Results

The results presented concern the pattern of the changing speeds of the volunteering participants, using the various postures under the scenarios studied. The acquired synthesized accelerations of the detected steps were processed in order to obtain the velocity of each volunteering participant. The data were then processed to identify any sudden changes in the average values, enabling the investigation of potential factors that influenced periodic velocity changes when the volunteering participants moved in a horizontal or vertical direction. 

### 3.1. Horizontal Velocity

#### 3.1.1. Changing Patterns and Possible Factors

##### Changing Patterns of Horizontal Velocity

The average steps under non-emergency were 151 (Male) and 162 (Female), while under emergency scenarios, they were 141 (Male) and 158 (Female), respectively; suggesting that under emergency people, especially male participants tend to have longer step lengths when moving in a quick manner. However, it is important to point out that the characteristics of the male participants showed that they were roughly 15 to 20 cm taller than the female participants hence they had comparatively longer stridden. 

**Figure 4:**
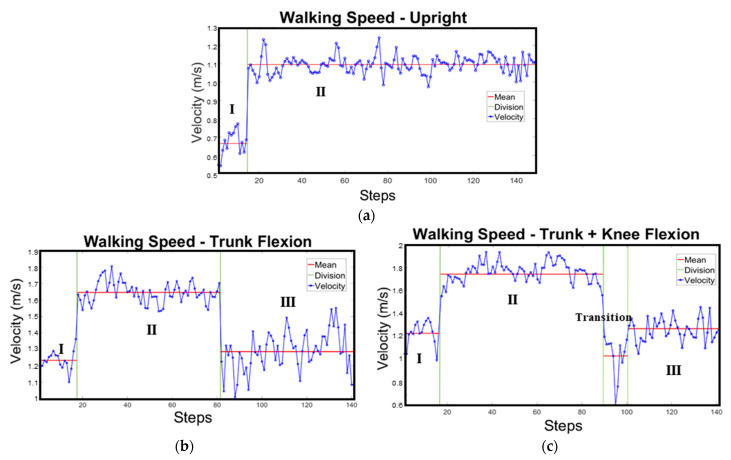
Instantaneous walking speed pattern of a participant using (**a**) Upright, (**b**) Trunk Flexion and (**c**) Trunk + Knee Flexion, under non-emergency and emergency scenarios (I~III represent the different stages of the walking process).

After analysing the collected data, it was found that the changing pattern of instantaneous walking velocities under non-emergency and emergency scenarios were similar for all participants, regardless of genders. Thus, for convenience, the data from one male participant has been used to explain the different stages of velocity changes under corresponding scenarios as shown in Figure 4. The subject used in example was randomly chosen out of all participants. Due to the noise in the instantaneous speeds as a result of the relatively high sensitivity of the smartphone-embedded accelerometer, average values have been used. This is also common with previous studies investigating the effects of postures on the velocities [22,23,27,28,32,33,35,49,60].

The instantaneous velocity changing pattern of the selected participant using upright posture under non-emergency scenarios could be found in Figure 4a. It is shown that the average walking speed can be divided into two stages, as Initial (I) and Comfortable (II) stages. This suggests that the participant needed an adaption process before adjusting to a preferred velocity. This also agrees with the social force theory of warming up period when adjusting to a comfortable a speed during movements [29]. The average speed during this initial stage was 0.66 m/s and during the comfortable stage it changed to 1.10 m/s.

Figure 4b shows the selected volunteering participant’s instantaneous walking speeds using Trunk-Flexion posture under emergency scenario. Three stages of the average walking velocities are shown here depicting Initial (I), Comfortable (II) and the Trunk-Flexion (III) stages. During the Initial (I), and Comfortable (II) stages, the selected volunteering participant used an upright posture. In stage I, the average velocity obtained was 1.22 m/s. In stage II, where the volunteering participant was upright walking comfortably, the average velocity shifted to 1.67 m/s. The average velocities in these two stages were both higher than those under non-emergency scenarios. This may be caused by panic leading to accelerated motion during the evacuation process [29,61]. Trunk-Flexion (Stage III) posture was used after passing the bending point (Figure 2) and resulted in a significant drop in the average velocity to about 1.30 m/s, comparatively close to the average velocity in Stage I. Figure 4c shows volunteering participants walking speeds using Trunk-Knee Flexion posture under emergency scenario. Here, four stages namely Initial (I), Comfortable (II), Trunk-Knee Flexion (III) and Transition stages are shown. Like in Figure 4b, the Initial (I), and Comfortable (II) stages represent volunteering participants in upright posture. The average velocities for Stage I and II here were like that in Figure 4b. During the Transition stage, there was a significant drop in velocity (1.02 m/s) before the Trunk-Knee Flexion stage (Stage III). This may be due to changes of in the centre of mass resulting from sudden knee bending before adapting to the new position [62,63]. As the inertial sensors were placed on the chest, it may have been sensitive to the changes in body posture [64]. At the Trunk-Knee Flexion stage, the average velocity increased to about 1.24 m/s, comparatively close to the average velocity in Stage I but still slightly slower than in Trunk-Flexion posture. 

The pattern of average walking velocities for all participants were like that observed for the single participant shown in Figure 4 and selected for the analysis. Table 3 provides a summary of the average speeds of all participants for different postures under the scenarios studied.

##### Analysis of Possible Factors for Horizontal Velocity Changes

From the above results, it can be observed that average male velocities in horizontal mode, under both scenarios using any posture type, were slightly higher than those recorded for the female participants. These results were consistent with previous studies indicating slower average speed for female participants than male participants [23,27,28], suggesting that gender could be a potential factor to influence evacuation [6,13,14]. As shown in Table 3, average speed in upright posture under non-emergency scenario was slightly slower than that under emergency scenarios. This suggest panic, as a psychological state, could be another factor that can accelerate motions during evacuation [29,61]. 

Along with psychological state and gender of evacuees, posture can also affect evacuation velocity changes during emergency situations. Evacuation posture be it upright or stoop cannot be ignored as it influences evacuation speed [23,27,28,35]. Orendurff et al. [65] found that hip and/or knee flexion can lead to evacuation speed reduction. As shown in Figure 4b in trunk-flexion posture, there was sudden transfer from a comfortable upright walking posture in Stage II to a stable state of Stage (III) with a lower speed. In trunk-knee flexion posture as shown in Figure 1, there was the need for a transition state which significantly declines the evacuation velocity to comparatively lower values before a rise to a stable state (Stage III), which has a higher velocity than the transition state but comparatively lower average velocity than Stage II. This may be because trunk-bending alone does not cause great changes in the centre of mass as knee-bending does [65]. Comparatively as can be seen in Table 3 and Figure 4b,c, trunk—flexion posture results in a higher average velocity than trunk-knee flexion. 

#### 3.1.2. Validation of Horizontal Velocity Results

In previous studies [9,22,23,27,28,32,33,35,38,39,41,43,45,49,52,53,60] where upright walking posture was the main focus, the average horizontal speed of participants was shown to vary greatly as can be seen in Table 4. In this study, measurements were carried out under different scenarios with focus on different postures and speeds. Most previous studies carried out experiments under 100% illumination, i.e., a well-lit environment either in daylight or full lights on but did not provide the illuminance levels. However, in this study, visibility was varied using 10% light transmission eyewear (Section 2.3). Some of the studies introduced more extreme conditions such as near-zero visibility [38,39,41,43,45,49,53], leading to a much slower average speed. 

A comparison of this study with previous studies is presented in Table 5 (the studies have been grouped based on illumination level, i.e., 100% and ≤10% visibility). It can be seen that the works by Cao et al. [27,28,32], Jeon et al. [49] and Juřík et al. [9] have similar experimental setups to this study except for their visibility factors and the additional factor of the volunteering participants’ familiarity of the building and its environment [12]. Thus, the following comparisons mainly focused on these works. 

The measured moving speed for an upright walker under non-emergency scenarios in this study was completed in accordance with theoretical values obtained from Helbing et al.’s social-force-based model [29]. In addition, as participants of this experiment were required to move under a dark mode with dark lens on, it was understandable that the acquired average velocity was comparatively slower than that collected under bright condition as in Cao et al. [27,28,32] and Seike et al. [45]. This shows that visibility is an important factor for velocity changes during evacuation [26,49,66]. Meanwhile, our results were comparatively higher than the upright velocity by Jeon et al. [49] (0.96 m/s) when moving under an environment of low visibility. This might due to the visibility conditions being better in this study as only dark mode with higher transparency without effects from smoke, while for the experiment by Jeon et al., they used a non-transparent eye patch with a visibility of only 5–10 m [49] (Table 5). This is also supported by the comparisons between other studies which have near-zero visibility experimental setups [38,39,41,43,45,53], with their relatively slower average speed. This also strengthens the argument that visibility could be a significant factor for walking velocities. 

The results were comparable to that of Seike et al. [45] where measurements were carried out under about 10 m visibility with slight amount of smoke. This suggests that the effects of slight smoke may be equivalent to the dark situation applied in this study while under heavy smoke, the average speed soon decreased [41,45]. Meanwhile, the acquired results were also similar to that of Juřík et al. [9], though the visibility conditions were different. This may be somehow related to the unfamiliarity to the environment of participants in Juřík et al. [9] and it implies that the unfamiliarity can have relatively similar effects as visibility on walking speed when moving in a horizontal direction. This finding is also supported by results from Anastasios et al. [39] as people were asked to evacuate in an unfamiliar tunnel with limited instructions, leading to slower speeds even under full-light. However, track length and age-diversity may be additional factors that may lead to fatigue during movements. 

Fridolf et al. [44] investigated visibility and walking speed and found that there was a linear relationship between walking speed with visibility when in an erect posture. The ratio between the acquired upright walking speed under dark mode in this study and that acquired under 100% illumination by Cao et al. [27] for both genders was about 0.87–0.88, which agreed with the previous findings. However, the ratio was not similar for different stoop postures, which was about 0.82 (trunk-flexion) and 0.78 (trunk-knee flexion), respectively, suggesting different stoop postures may have additional slowing effects during the evacuation process. 

### 3.2. Vertical Velocity

#### 3.2.1. Changing Patterns and Possible Factors

##### Changing Pattern of Vertical Velocity

The average steps during the descent under the non-emergency scenarios were 170 (male) and 176 (female), while under the emergency scenarios, the average steps were 165 (male) and 174 (female), with a fixed 78 steps on the stairs and other steps on the floors or transition areas between staircases (Figure 3b). Like the horizontal experiments, the male participants had longer stridden lengths under emergency scenarios. However, in the vertical movements, it was slightly affected by the fixed steps on the staircases. 

When participants were moving down staircases, this could be treated as a repeated height-change pattern, and these participants are able to keep their desirable speed when descending floors [51]. On the other hand, as previously mentioned in the horizontal movements (Section 3.1.1), the instantaneous speeds could be affected by the high-noise smartphone-based data. Thus, it is common to use one’s average walking speed in order to analyse the moving patterns when moving downstairs [51,55,56,57,58,59,67]. 

**Figure 5:**
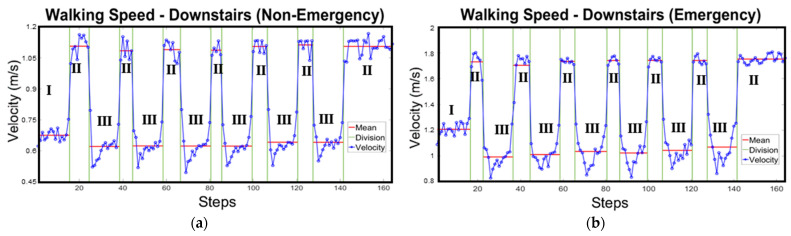
The instantaneous walking speed changing patterns of one subject under non-emergency (**a**) and emergency scenarios (**b**) (I~III represent the different stages of the vertical movements).

Like the horizontal movements, all participants shared a similar pattern of average walking speed differences when moving downstairs, regardless of the gender or scenario. Only the upright posture was used during the vertical experiments, due to potential fall risks. Once again, for convenience, data from a randomly selected participant shown in Figure 5 are used to analyse the different stages of walking velocities. 

Figure 5 shows the selected volunteering participant’s instantaneous walking speeds using an upright posture under the different scenarios. The entire process could be divided into three stages: Initial (I), Comfortable (II), and Staircase Movements (III). When moving in the horizontal direction, the walking velocity changes were similar to that of the horizontal experiments (Section 3.1.1). However, when approaching the staircase, the average walking speed decreases to a relatively lower speed (Stage III), though the instantaneous speed does not decrease at the first step. In fact, the changing process for instantaneous speed when moving downstairs is more likely to be a left-centred “U-Shape” (Figure 5b) or “V-Shape” (Figure 5a), involving a process of a self-adaption, which involves an initial slowing down for a few steps and a gradual speeding up when approaching floors or transition areas. The average moving velocities in these transition areas resumed to the comfortable state (Stage II), with the specific values depending on the corresponding scenarios. Moreover, it could be observed that the average velocities on the different staircases (besides the floor transition areas) were similar, and were also slower than whose measured for the horizontal movements (Figure 5).

The patterns of the average vertical moving velocity changes for all participants were similar to those of the single participant. Table 6 presents a summary of the average speeds for Stages II and III participants used staircases within both the scenarios in an upright position. 

##### Analysis of Possible Factors for Vertical Velocity Changes

Similar to the horizontal measurements, panic appears to influence movement velocity [29,61]. The only difference between non-emergency (Figure 5a) and emergency scenarios (Figure 5b) was the lower average values at different stages (Table 6). For instance, the instantaneous velocity range of the selected participant was between 0.45–1.2 m/s (Figure 5a) under non-emergency conditions, while, for emergency conditions, it increased to 0.8–2 m/s. 

According to the results, gender was prominent in the vertical speed differences. The average vertical velocities were about 0.64 m/s (male) and 0.58 m/s (female) under non-emergency modes, while, under emergency conditions, the average values were about 0.96 m/s (male) and 0.86 m/s (female). Though male participants had a slightly higher average value than their female counterparts, the difference was not as significant as that in the horizontal movements. Considering the calculated high standard deviation, the average velocities could be treated as similar for both genders, as is consistent with previous work by Proulx et al. [38]. 

Comparing the horizontal speed in an erect posture, it was observed that the average moving velocity on the staircases was slower than that on the floor. The results show that the vertical velocity was about 42.48% slower than that recorded for the horizontal movement under non-emergency scenarios. This reduction was comparatively higher under emergency scenarios for the horizontal movement, where the average was 45.34%. 

#### 3.2.2. Validation of Vertical Velocity Results

In previous studies, the experiments were conducted under a well-lit environment, which was either in daylight or with lights fully on. However, the majority of them did not provide detailed values of the lighting conditions. Thus, this study just simply treated all these lighting setups as 100% illumination, while this study used eyeglasses with a 10% light transmission (Section 2.3), which is much closer to the lighting condition in Proulx et al. [50] (approximately 74 lux). The detailed experimental setups can be seen in Table 7. 

Based on this available information, the experimental setups in this study were closer to the works by Fang et al. [56], Huo et al. [67], Zeng et al. [51], and Proulx et al. [50]. The work from Juřík et al. [9] does not provide the details of the riser and thread of the stairs, therefore, this work is not an ideal option for comparison, though the floor level and the participants’ characteristics were similar. Hence, the comparison focused mainly on four works previously mentioned, the latter one from Juřík et al. [9] is compared separately. The works by Chen et al. [58] and Lu et al. [59] introduced some extreme conditions, such as more floors and 0% visibility, which are also used as part of the supporting evidence in later comparisons. 

As shown in Table 8, previous studies have investigated average vertical speeds when moving downstairs with different thread and riser ratios, under different illumination conditions. These studies show vertical movement speeds, ranging between 0.62 m/s and 1.25 m/s [51,55,56,57,67]. The thread and riser ratios of the staircases in this study were closest to those found in Fang et al. [56]. However, the vertical speed in this study was comparatively higher under less illumination. This may be that the average velocity obtained by Fang et al. [56] was based on a building with more storeys (8 floors) and comparatively older participants (Table 7). This also shows the effect of the participants’ age on the evacuation speed, as participants in this present study were all young adults aged between 21–23. This is also consistent with findings by Proulx et al. [38], where similar illumination conditions (0.72 m/s, 10% illumination), as well as lower storeys and similar average ages were used. Compared to the work of Huo et al. [67] (0.85 m/s), also with a similar age range of participants, more floors in the building influences the vertical moving speed, a point also acknowledged by Ma et al. [57]. This is also supported by the results from Zeng et al. [51] (1.25 m/s, 100% illumination), where the study was conducted in a building with fewer storeys, and Chen et al. [58] (1.15 m/s, 100% illumination), where there were also more storeys. These two studies were both conducted under 100% visibility, but the latter used twenty floors, while the former used only six floors. 

Like the horizontal movements, the level of illumination impacts the vertical moving velocity. The vertical velocity obtained in this study was comparatively lower than the average obtained by Zeng et al. [51] (under 12% illumination), but was higher than all averages under 0% illumination [50,51,58,59], as shown in Table 8. On the other hand, unfamiliar buildings and their environments may not have similar effects as visibility when moving vertically downwards as it does for horizontally. This is because, unlike horizontal movements where one would look for exits on a planar level, moving downwards during evacuations are usually directed towards the final exits on the ground floor. This is evident from the similar average speeds obtained by Juřík et al. [9] when compared to those in Zeng et al. [51], possibly as a result of both studies having similar stairway setups. Based on this assumption and the speed comparison between our present study and that of Juřík et al. [9], it can be observed that the low visibility resulted in a lower walking speed. Thus, when moving vertically downwards during evacuations, visibility appears to be a more important factor than the familiarity with the building and its environment, and this is evident in the walking speeds recorded. However, a longer track can be seen to have more impact than low visibility, particularly when comparing the results between that of Lu et al. [59] (0% visibility) and other studies using less than 10% visibility [50,58]. The study by Lu et al. [59] used fewer storeys and a lower visibility, but had higher average speeds than the other two studies. However, the results from Lu et al. [59] were still comparatively slower than our results with slightly better visibility conditions. This implies that fatigue is more impactful under some extreme visibility (less than 10 m) conditions, making this factor worthy of further investigation. 

## 4. Discussion

This study has identified a “warming-up” process of upright walking, i.e., speed adjustment [15], regardless of gender and emergency conditions. This is supported by the theory of social force, as raised by Helbing et al. [29], where people would make themselves accustomed to a more comfortable speed of moving after moving for a while. The horizontal velocity under a relaxed state was similar to the theorical results obtained in previous studies [29,60]. However, as the velocities in this study were obtained under low-visibility (10% illumination), it suggests that, under 100% illumination, they should be comparatively higher [26,49,66]. This implies that visibility during evacuations is important for a quick and efficient exit to safety. 

Panic during evacuations, a psychological factor, can to some extent also be affected by the visibility. In previous studies, it is reported that people tend to move faster during emergencies when there is good visibility [29,61]. Typical walking speeds in a relaxed manner have previously been reported to be about 63.64–66% faster [22,30]. However, in this study, the velocity increased in a similar manner ranging between 54.39–60%, due to the lower visibility. From Jeon et al. [49], the better the visibility, the more likelihood there is of smaller velocity differences between the vertical and horizontal movements. 

Gender was also found to have some effects on the walking speeds when participants moved in the horizontal direction. Cao et al. [27,28,32] and Kady and Davis [23] found that females tend to move comparatively slower than their male counterparts during an evacuation scenario. However, this effect was observed to be attenuated by changing postures into stooped walking, where the difference in speeds between the two genders was small. Comparing the horizontal speed between genders under the emergency scenarios, female participants were about 10.80% slower than male participants with an upright posture, while, for the stooped posture, female participants were about 8.45% slower than the male participants. In previous studies, the reduction in the difference in speeds between the two genders using different postures was in the range of 3.44–10%, comparatively higher than what has been observed in this study. This may be as a result of the fact that this study simulated a low-visibility environment, impacting the evacuation speeds of participants [49]. However, this effect was nearly negligible when participants moved vertically, consistent with the findings from Proulx et al. [38].

The application of different postures played a significant role under the emergency scenarios. When transferring from the upright posture to either type of the stoop walking postures, the speed from both genders were observed to decrease. Additionally, this change of speed appeared nearly simultaneously with the posture changing during the process of moving horizontally under emergency conditions. This also suggests that posture transformation has a direct impact. For the trunk flexion posture, the average speed of male participants reduced by 19.32%, while that of the female participants reduced by 17.20%. On the other hand, the average speed using trunk–knee flexion reduced by 21.02% and 20.38% for males and females, respectively. This suggests that the latter posture, trunk–knee flexion, could lead to a comparatively higher speed reduction during evacuation. Therefore, where the stooped posture is required for evacuation, the trunk flexion posture might be more suitable if the speed of evacuation is paramount. Thus, we recommend the trunk flexion posture when stooped walking is adopted in evacuation procedures. 

In addition, the unique transition phase from the trunk–knee flexion posture could be used as a plausible feature for posture identification as this only happened when transferring to this specific posture. This was validated by the repeated experiments and surveillance data in this study. The accelerometer used had a slightly higher sensitivity and detected the beginning of the posture changes; this was due to the fact that the change of the personal height was sometimes not significant enough for posture detection. 

As mentioned earlier in the results, the visibility effect on the moving speed could be represented as a linear relationship [44]. Thus, the ratio of the velocity with and without visual impact is supposed to be constant if there are no changes in visibility. This relationship, however, could be affected by different postures. The ratio between the affected velocity and the unaffected velocity in a previous study by Cao et al. [27] was a constant for both genders when using the erect posture (≈0.88). However, this ratio decreased to 0.82 (trunk flexion) and 0.78 (trunk–knee flexion), respectively, when different stoop postures were adopted during the simulated scenarios. This also supported the previous hypothesis that the trunk flexion posture is a better choice for speed maintenance when required to move under a lower height. 

Moreover, compared to Juřík et al. [9], the unfamiliarity may have similar effects as low-visibility when moving horizontally. However, longer moving distances could be an impacting factor when moving horizontally. This can be observed from the average horizontal speed obtained by Anastasios et al. [39], and when compared to that of other previous studies [27,28,32], where a slower average speed was recorded. This effect can almost be neglected when moving vertically downwards, as shown in Table 8, where Zeng et al. [51] and Juřík et al. [9] had similar vertical velocities under 100% visibility with participants unfamiliar with the building and its environment. In addition, the number of storeys can have more impact than visibility when moving under extreme conditions (<10% visibility), as shown in the results from Lu et al. [59] (0% visibility) and other studies using less than 10% visibility [50,58]. The findings in this study, supported by other studies, highlight that further decreases in the visibility do not significantly cause a reduction in moving speed, regardless of whether moving horizontally [38] or vertically [45]. 

In this study, moving in the vertical direction has been found to lead to a roughly 41.81–45.45% decrease in speed when compared to horizontal movements, regardless of the urgency of the conditions or gender (Table 5). The speed under emergency conditions compared to non-emergency conditions increased by about 53.92–60% for horizontal movements, while in the vertical direction, it increased by about 48.28–50%. In terms of gender, this speed reduction was found to be more significant for male participants, as they experienced a 10% reduction, while female participants experienced roughly a 5.64% reduction in speed. This also agrees with the findings from Cao et al. [27,28,32] (Table 3). 

### Limitations of Study

Although this study has been validated against reputable peer reviewed findings, there are still some limitations worth consideration for further improvements. 

Firstly, only one level of the low illumination condition was used to simulate an environment of relatively low visibility using eyeglasses (goggles) of 10% light transmissibility. Hence, participants’ speeds under 100% illumination were not obtained for comparison. Here, we recommend that future studies include different levels of illuminations that mimic varying real-life emergency situations (including a full-light condition) to allow for a more comprehensive analysis. 

Secondly, the emergency scenarios were created by instructions from instructors and not by fire alarms in actual fire events, both of which have different psychological effects on the participants, which would explain the results obtained. In future studies, similar experiments could be conducted under a fire drill with more participants, as the evacuee density is also an important factor which causes panic and thus impacts the walking velocities under the situation of good visibility. While in a low-visibility environment, the effect of pedestrian density on moving speeds may not be as significant as that under the situation of 100% illumination, regardless of horizontal [53] or vertical [58,59] movement. However, it is still worthwhile to further investigate crowd density under varying visibility conditions in order to analyse pedestrian behaviours in emergency situations.

Another limitation here is to do with the evaluation of the psychological (panic) state of the participants during emergency calls. Cao et al. [27,28,32] involved the measurements of average heart rates to quantify the degree of panic in order to evaluate the psychological impacts (panic state) of participants in their studies. In this present study, we were unable to evaluate this; therefore, the extent of the participants’ panic state during the different stooped postures was not successfully ascertained. 

Furthermore, the volunteering participants for this experiment were active young undergraduate students who were familiar with the building and its environment. Therefore, other factors related to the building occupants’ characteristics, such as varying age demographics, varying abilities, and body masses were not considered [9,10,15]. However, the credibility of the present work is unaffected, as this demographic of people are large occupiers of buildings, typically in colleges or the higher education sector worldwide. Evacuations would be required in such buildings during emergencies. However, we recommend future studies to consider varying characteristics in this demographic of people and include faculty members of varying ages, abilities, and body masses, making the procedure much more representative and realistic. Scenarios involving participants unfamiliar with the building and its environment can also be considered. 

Additionally, due to the risk of injury to participants, the stooped postures were not used in the vertical direction. For this reason, we were unable to evaluate the impact of these postures in the vertical direction. Moreover, as the crawling was not a focus of this study, it was not considered in this study. Future studies can consider several other postures for diverse volunteers in order to mimic real evacuations during real fire emergencies. Also, as the route designed for the speed adaption experiment had a corner, this affected the walking speeds of the participants on approaching these corners; thus, our results should be seen as factoring this in. Upon vertical descent, although there were no corners for participants to turn as with the horizontal movements, there were directional changes on each floor landing, which also impacted the participant speeds during the evacuation simulation. 

Finally, the uncertainty with the measurement instruments, along with its syncing with the beginning of the measurements and data noise potentially led to some errors in the results. Also, the sensor location and sensor sensitivity during the changes in posture potentially influenced the results obtained. In future studies, these can be improved by having more testing on different sensor locations on the body with more smartphone-models involved.

## 5. Conclusions

Two different stooped postures, trunk flexion and trunk–knee flexion, and an upright posture have been investigated in how they affect evacuation walking speeds under low visibility (10% light transmission) in the vertical and horizontal directions, respectively. The investigations were carried out under emergency and non-emergency scenarios, using 3D PVINS, a vision-assisted system for indoor positioning, with participants familiar with the building and its environment. The results show the following:

1. In the horizontal walking direction, there were variations in walking velocities between genders, with male speeds being comparatively higher than those of their female counterparts. The average speed in the upright posture under non-emergency scenarios for male participants was 0.66 m/s, while for female participants, it was 0.60 m/s. In the upright posture under emergency scenarios, the average speed for male participants ranged between 1.20 and 1.76 m/s, while for female participants, it ranged between 1.02 and 1.57 m/s. When participants used the trunk flexion posture, the average speed for male participants was 1.42 m/s, while for female participants, it was 1.30 m/s. In the trunk–knee flexion posture, the average speed for male participants ranged between 1.01 and 1.35 m/s, while for female participants, it ranged between 0.96 and 1.25 m/s.
2. In the vertical walking direction, there were also variations in walking velocities between genders, with female participants having comparatively lower velocities. Here, in the upright posture under non-emergency scenarios, male participants achieved an average speed of 1.10 m/s on the horizontal floor, and 0.64 m/s when descending downstairs to the exit. For female participants under the same scenario, average speeds of 1.02 m/s on the horizontal floor and 0.58 m/s when descending to the exit were achieved. Under the emergency scenario, the speeds of both genders increased. Here, the average speed for male participants was 1.76 m/s on the horizontal floor and 0.96 m/s when descending the stairs. For female participants, the average speed was 1.57 m/s on the horizontal floor and 0.86 m/s when descending the stairs to the exit.
3. The speeds between the horizontal direction and the vertical direction in the upright posture also varied. It was observed that the average moving velocity of participants on staircases were comparatively slower than on the horizontal floor. Here, the average vertical velocity was found to be about 42.48% slower than the horizontal velocity when participants moved under non-emergency scenarios. Under emergency scenarios, the average vertical velocity was found to be about 45.34% below the horizontal velocity.
4. When transitioning from the upright posture to any of the stooped walking postures, the speed of both genders decreased. When transitioning from the upright to the trunk flexion posture, the average speed of male participants reduced by 19.32%, while for female participants, it reduced by 17.20%. When transitioning from the upright to trunk–knee flexion, the average speed reduced by 21.02% for male participants and 20.38% for female participants.

Overall, our findings show that moving directions caused about a 43.90% speed reduction, while the posture accounted for over 17% of speed changes amongst the participants involved in this study. Gender, on the other hand, accounted for less than 10% of the differences in speed. The speeds of the participants under the emergency scenario increased roughly by 53.92–60% in the horizontal direction, while in the vertical direction, it increased by about 48.28–50% when compared to the participants’ speeds under non-emergency scenarios. 

From the results, the trunk flexion posture led to faster evacuation moving speeds, making this worth investigating further in fire evacuation simulations and related fire drill trainings. Here, the consideration of diverse groups of people during these simulations and drills would be crucial. 

The collected data from the study could also be further utilised as referential data for the agent-based modelling of evacuations in similar buildings (office or teaching buildings). This could be useful in a relevant database as a benchmark when testing the function of newly designed buildings on the aspect of safe evacuations.

In future studies, we recommend evacuee density is considered, along with panic (evacuees psychological state) and walking velocities using the different postures. Also, such studies could be calibrated to involve varied illuminances in order to reflect real fire evacuation conditions. 

## Figures and Tables

**Table 1:** The details of experimental sensors.

Instrument	Specification	MAE * of Measurement (m)	Experiment	Calibration
Accelerator	embedded sensors in iPhone 7 Plus ^1^/Huawei Mate 8 ^2^	0.16	Horizontal and Vertical	Calibrated using camera
	Sample Rate: 100 Hz			
Barometer	embedded sensor in iPhone 7 plus ^1^/Huawei Mate 8 ^2^	0.5	Vertical Only	Self-calibrated
	Sample Rate: 1 Hz			
Camera	Resolution: 680 × 540	0.06	Horizontal Only	Self-calibrated
	FOV: 27°			
	Detection rate: 17 Hz			

^1^ Made from Apple Inc, California, USA; ^2^ Made from Huawei Technologies Co., Ltd., Shenzhen, China; * MAE: Mean Average Error.

**Table 3:** The Velocity (Mean ± SD *) of Different Postures for Both Genders under Non-Emergency and Emergency Scenarios.

	Non-Emergency Scenario (m/s)		Emergency Scenario (m/s)				
Posture	Upright Walking		Upright Walking		Stoop Walking A		Stoop Walking B
State	Initial (I)	Comfortable (II)	Initial (I)	Comfortable (II)	Trunk-Flexion (III)	Transition (IV)	Trunk-Knee Flexion (III)
Male	0.66 ± 0.30	1.10 ± 0.32	1.20 ± 0.40	1.76 ± 0.32	1.42 ± 0.24	1.01 ± 0.42	1.35 ± 0.22
Female	0.60 ± 0.39	1.03 ± 0.31	1.02 ± 0.39	1.57 ± 0.39	1.30 ± 0.25	0.96 ± 0.40	1.25 ± 0.20

* SD represents the standard deviation.

**Table 4:** Comparison of Average Horizontal Velocity using Different Postures under Non-Emergency and Emergency Scenarios under various illumination level.

Illumination			Non-Emergency Scenario (m/s)				Emergency Scenario (m/s)			
	Posture		Initial Upright Walking (I)		Comfortable Upright Walking (II)		Upright Walking		Stoop Walking	
		Gender	Male	Female	Male	Female	Male	Female	Male	Female
	Research									
100%	Helbing et al. [29]		0.60	0.60	1.00	1.00	1.50	1.50		
	Trivedi and Rao [61]		0.60	0.60	1.00	1.00	1.50	1.50		
	Li and Chow [17]		0.60	0.60			1.30	1.30		
	Ugwitz et al. [52]				1.19	1.19	2.22	2.09		
	Gallagher et al. [35]						1.33	1.33	1.01	1.01
	Nagai et al. [33]						1.20	1.20		
	Muhdi et al. [22]				1.32	1.32	2.16	2.16		
	Hurley et al. [60]		0.60	0.60	1.01	1.01	1.25	1.25		
	Kady and Davis [23]						1.74	1.63		
	Jeon et al. [49]						0.96	0.96		
	Cao et al. [28,32]						2.28	1.57	2.13	1.54
	Cao et al. [27]						2.00	1.80	1.70	1.70
	Juřík et al. [9]				1.19	1.19	1.82	1.78		
	Xie et al. [53]						0.78	0.78		
	Seike et al. [45]						2.03	2.03		
≤10%	Seike et al. [45]						1.74	1.74		
	**This Study**		**0.66**	**0.60**	**1.10**	**1.01**	**1.76**	**1.57**	**1.42**	**1.30**
	Anastasios et al. [39]						1.14	1.14		
	Xie et al. [53]						0.54	0.54		
	Xue et al. [43]				0.4	0.4	0.6	0.6		
	Cao et al. [38]				1.32	1.32				
	Cao et al. [38]				0.61	0.61				
	Anastasios et al. [39]						1.05	1.05		
	Seike et al. [41]						0.52	0.46		

**Table 5:** Summary of the Experimental Setups of Horizontal Movements in Previous Studies.

	Parameters	No. of Participants (Male/Female)	Age	Height (cm)	Weight (kg)	BMI	Track Length (m)	Illumination	Simulation/ Experiment
Research									
Helbing et al. [29]		80 (N/A)	N/A (young adults)	N/A	80	N/A	15.0	100%	Simulation
Trivedi and Rao [61]		160 (N/A)	N/A (young adults)	N/A	65	N/A	35.0	100%	Simulation
Li and Chow [17]		82 (N/A)	N/A (young adults)	N/A	N/A	N/A	15.2	100%	Simulation
Ugwitz et al. [52]		20 (10 M/10 F)	20–26	N/A	N/A	N/A	161.52	100%	Simulation
Gallagher et al. [35]		9 (6 M/3 F)	35–52	160.4~175.6	59.1~80.3	20.2~28.2	N/A	100%	Experiment
Nagai et al. [33]		60 (N/A)	N/A (college students)	N/A	N/A	N/A	6.0	100%	Experiment
Muhdi et al. [22]		26 (18 M/8 F)	N/A (college students)	N/A	N/A	21.6~26.0	30.5	100%	Experiment
Hurley et al. [60]		6 (N/A)	N/A (adults)	N/A	N/A	N/A	18.0	100%	Experiment
Kady and Davis [23]		18 (9 M/9 F)	19–29	N/A	N/A	18.5~30.0	30.5	100%	Experiment
Jeon et al. [49]		31 (15 M/16 F)	35	165.3	N/A	N/A	199.9	N/A (5~10 m visibility)	Experiment
Cao et al. [28,32]		24 (12 M/12 F)	23–27	161.8~180.7	52.3~79.6	19.8~24.8	91.44	100%	Experiment
Cao et al. [27]		24 (12 M/12 F)	N/A (college students)	165.0~175.0	N/A	18.5~30.0	45.72	100%	Experiment
Juřík et al. [9]		35 (15 M/10 F)	20–26	N/A	N/A	N/A	183.91	100%	Experiment
Xie et al. [53]		36 (27 M/9 F)	N/A (college students)	N/A	N/A	N/A	6.96	N/A (6~10 m visibility), 100%	Experiment
Xue et al. [43]		30 (15 M/15 F)	19–27	N/A	N/A	N/A	10	≈0%	Experiment
Cao et al. [38]		41 (23 M/18 F)	18~23	160~185	40~80	15.6~23.4	16	0%, 100%	Experiment
Seike et al. [45]		184 (137 M/47 F)	18–82	N/A	N/A	N/A	150	N/A (2~10 m visibility)	Experiment
**This Study**		**20 (10 M/10 F)**	**21~23**	**160~180**	**50~75**	**19.9~24.2**	**92.75**	**10%**	**Experiment**
Anastasios et al. [39]		20 (14 M/5 F)	15~68	N/A	N/A	N/A	164.5	≈0%, 100%	Experiment
Seike et al. [41]		30 (17 M/13 F)	18~71	N/A	N/A	N/A	488	0%	Experiment

**Table 6:** The velocity (Mean ± SD) when in an upright position under non-emergency and emergency scenarios.

	Scenarios	Non-Emergency Scenario (m/s)		Emergency Scenario (m/s)	
Gender		Floor (II)	Staircase (III)	Floor (II)	Staircase (III)
Male		1.10 ± 0.32	0.64 ± 0.33	1.76 ± 0.32	0.96 ± 0.28
Female		1.02 ± 0.31	0.58 ± 0.32	1.57 ± 0.39	0.86 ± 0.26

**Table 7:** Summary of the environmental setups of horizontal movements in previous studies.

	Parameters	No. of Participants (Male/Female)	Age	Riser (cm)	Tread (cm)	BMI	Floor Layer	Illumination	Simulation/ Experiment
Research									
Nelson and Mowrer [55]		8 (N/A)	N/A (young adults)	16.51 17.78 19.05	33.02 30.48 29.04 25.04	N/A	N/A	100%	Simulation
Fang et al. [56]		6 (4 M/2 F)	21–62	16.5	28.5	N/A	8	100%	Experiment
Huo et al. [67]		73 (53 M/20 F)	23.1	15.0	27.5	20.90	9	100%	Experiment
Ma et al. [57]		177 (108 M/69 F)	21–62	15.0	28.5	N/A	12–17	100%	Experiment
Zeng et al. [51]		38 (19 M/19 F)	N/A (college students)	15.0	27.5	N/A	6 (9th to 3rd floor)	0%, 12%, 100%	Experiment
Juřík et al. [9]		35 (15 M/10 F)	20–26	N/A	N/A	N/A	4	100%	Experiment
Chen et al. [58]		30 (15 F/15 M)	17–22	16.0	26.0	20.02–2.3	20	0%, <10%, 100%	Experiment
**This Study**		20 (10 M/10 F)	21–23	16.0	29.0	19.9–24.2	4	10%	Experiment
Lu et al. [59]		48 (28 M/22 F)	23.4	17.0	33.0	19.6–21.6	2	0%	Experiment
Proulx et al. [50]		39, 77 (N/A)	20–60	N/A	25	N/A	9–11	0%, ≤10% (74 lux)	Experiment

**Table 8:** Comparison of average vertical velocity when walking upright under emergency scenarios.

Research	Riser (cm)	Tread (cm)	Illumination (%)	Velocity (m/s)
Nelson and Mowrer [51,55]	19.05	25.04	100%	0.85
Nelson and Mowrer [55]	17.78	29.94	100%	0.95
Nelson and Mowrer [55]	16.51	30.48	100%	1.0
Nelson and Mowrer [55]	16.51	33.02	100%	1.05
Fang et al. [56]	16.5	28.5	100%	0.81 ± 0.13
Huo et al. [67]	15.0	27.5	100%	0.85
Ma et al. [57]	15.0	28.5	100%	0.62
Zeng et al. [51]	15.0	27.5	100%	1.25 ± 0.28
Chen et al. [58]	16.0	26.0	100%	1.15 ± 0.22
Juřík et al. [9]	/	/	100%	1.24 ± 0.68
Zeng et al. [51]	15.0	27.5	12%	1.12 ± 0.28
**This Study**	**16.0**	**29.0**	**10%**	**0.92 ± 0.28**
Chen et al. [58]	16.0	26.0	<10%	0.59 ± 0.15
Proulx et al. [50]	/	25	≤10% (74 lux)	0.72
Lu et al. [59]	17.0	33.0	0%	0.87 ± 0.67
Zeng et al. [51]	15.0	27.5	0%	0.50 ± 0.14
Chen et al. [58]	16.0	26.0	0%	0.50 ± 0.13
Proulx et al. [50]	/	25	0%	0.57

## Data Availability

The data presented in this study are available on request from the corresponding author. The data are not publicly available due to the involvement of personal data and data sharing is not listed in the informed consent.

## Nomenclature

**Name**	**Description**
TET	Total Evacuation Time
BMI	Body Mass Index
PVINS	Passive Vision-aided Inertial System
TSW	Trunk Flexion Stoop Walking
TKSW	Trunk–Knee Flexion Stoop Walking
$H_{P}$	Body height of the person
${SL}_{i}$	Step length of *i*-th step
$v_{h}\left(i\right)$	Instantaneous velocities of *i*-th step in horizontal direction
$v_{v}\left(i\right)$	Instantaneous velocities of *i*-th step in vertical direction
$\bar{v_{h}}$	Average horizontal speed
$\bar{v_{v}}$	Average vertical speed
$n_{j}$, $n_{k}$	Number of steps in Stage j or k
